# Analysis of genomic alterations in cancer associated human pancreatic stellate cells

**DOI:** 10.1038/s41598-022-17748-1

**Published:** 2022-08-08

**Authors:** Viktoria Böker, Johanna Häußler, Jenny Baumann, Yoshiaki Sunami, Bogusz Trojanowicz, Bernadette Harwardt, Kathrin Hammje, Nadine von Auw, Mert Erkan, Knut Krohn, Jörg Kleeff

**Affiliations:** 1 Department of Visceral, Thoracic and Vascular Surgery, University Medical Center Carl Gustav Carus Dresden, 01307 Dresden, Germany; 2 Department of Visceral, Vascular and Endocrine Surgery, Martin-Luther-University Halle-Wittenberg, University Medical Center Halle, 06120 Halle, Germany; 3 Acibadem University Hospital Atakent, Istanbul, Turkey; 4 Core Unit DNA im SIKT, Medical Faculty, University Leipzig, 04103 Leipzig, Germany; 5 Department of Visceral, Vascular and Endocrine Surgery, University Hospital Halle, Martin-Luther-University Halle-Wittenberg, University Medical Center Halle, Ernst-Grube-Straße 40, 06120 Halle, Germany

**Keywords:** Cancer, Cell biology, Computational biology and bioinformatics, Genetics, Diseases, Medical research, Oncology, Pathogenesis

## Abstract

Pancreatic stellate cells (PSCs) constitute important cells of the pancreatic microenvironment and their close interaction with cancer cells is important in pancreatic cancer. It is currently not known whether PSCs accumulate genetic alterations that contribute to tumor biology. Our aim was to analyze genetic alterations in cancer associated PSCs. PSC DNA was matched to DNA isolated from pancreatic cancer patients’ blood (*n* = 5) and analyzed by Next-Generation Sequencing (NGS). Bioinformatic analysis was performed using the GATK software and pathogenicity prediction scores. Sanger sequencing was carried out to verify specific genetic alterations in a larger panel of PSCs (*n* = 50). NGS and GATK analysis identified on average 26 single nucleotide variants in PSC DNA as compared to the matched blood DNA that could be visualized with the Integrative Genomics Viewer. The absence of PDAC driver mutations (*KRAS*, *p53*, *p16/INK4a*, *SMAD4*) confirmed that PSC isolations were not contaminated with cancer cells. After filtering the variants, using different pathogenicity scores, ten genes were identified (*SERPINB2*, *CNTNAP4*, *DENND4B*, *DPP4*, *FGFBP2*, *MIGA2*, *POLE*, *SNRNP40*, *TOP2B,* and *ZDHHC18)* in single samples and confirmed by Sanger sequencing. As a proof of concept, functional analysis using control and *SERPINB2* knock-out fibroblasts revealed functional effects on growth, migration, and collagen contraction. In conclusion, PSC DNA exhibit a substantial amount of single nucleotide variants that might have functional effects potentially contributing to tumor aggressiveness.

## Introduction

Pancreatic cancer is associated with a high mortality rate, which has not changed over the last decades^1^. There are no specific symptoms which is one of the reasons why patients get their diagnosis in advanced stages. The only treatment that offers long term survival and the chance of cure in a small percentage of patients is surgical resection combined with adjuvant chemotherapy (5-year survival rate ~ 15–25%^2^), which is often not possible, because of the aggressive tumor growth, including local and/or distant metastases. In unresectable cases, response to the conventional chemotherapeutics although improving recently, is still not satisfactory.

Our understanding of the molecular biology of pancreatic cancer and its driver mutations has significantly increased. Further, there is a trend focusing on the microenvironment and its cellular components to further analyse the mechanisms supporting the development of pancreatic cancer.

Among the stromal cells of great interest are pancreatic stellate cells (PSCs), which share similarities with hepatic stellate cells and are present in the dense stroma of pancreatic cancer. However, their exact role in the processes of tumorigenesis—tumor supportive versus tumor restraining—is not clear. Activation of these cells is related to the loss of accumulated lipid droplets and vitamin A, and phenotypic changes such as myofibroblastic-like morphology^3^.

PSCs can be subdivided in different groups. For example, PSCs can be classified concerning their contractile phenotype as myCAFs, a subgroup of cancer-associated fibroblasts (CAFs) and iCAFs which show an immunomodulating secretome^4^.

These activated PSCs are able to produce large amounts of collagens, e.g. type type I and III, laminin and fibronectin^5^ and activation of stellate cells occurs in growth factor-, cytokine- and/or oxidative stress dependent manner^6^.

Tumor cells themselves may produce various growth factors such as transforming growth factor βs (TGF-βs), vascular endothelial growth factor (VEGF) and platelet-derived growth factor (PDGF), which are known to activate PSCs^6^. PSCs not only interact with tumor cells, but they also interact with many different cells in the microenvironment like islet cells, neuronal cells, endothelial cells and immune cells^1^. They can increase the proliferation and duct formation of endothelial cells. Furthermore, PSCs can also affect the immune system by reducing T-cell activity in tumors, increasing mast cell activity and elevating the myeloid suppressor cell migration into the tumor. All of these functions directly or indirectly influence tumor cells. The production of massive amounts of extracellular matrix proteins contribute to the generation of a hypoxic environment that can also support tumor growth^1,6^.

A detailed characterization and analysis of the stellate cells is crucial for the understanding of the mechanisms of how tumor and stroma cells interact with each other^3^.

It is currently not clear whether PSCs functions are only regulated at a transcriptional, epigenetic level, or whether there are also genetic alterations that might provide PSCs with an advantage in the tumor environment and therefore indirectly influence tumor growth. Building upon our previously published work showing that cancer-associated PSC display transcriptional differences as compared to those originating from chronic pancreatitis tissues^7^, this study aims to identify potential genetic alterations in cancer-associated human PSCs which might have an effect on PSC function in pancreatic cancer.

## Material and methods

### Patients

Pancreatic cancer tissue samples (*n* = 50) were collected from patients treated from June 2017 to December 2019 in the Department of Surgery of the Martin-Luther University Halle, Germany. Pathological diagnosis of pancreatic ductal adenocarcinoma and chronic pancreatitis in tissue sections was confirmed by haematoxylin and eosin staining. All tissues were fresh isolated in cell culture medium. 1 × 10^6^ cells were stored at − 150 °C for further proceedings in 90% FBS 10% DMSO. Total DNA was extracted from all samples using the QIAamp DNA Blood Mini Kit according to the manufacturer´s instructions.

Blood samples for DNA isolation (250 µl EDTA blood) were obtained from the same patients. The study was approved by the ethical committee of the Martin-Luther University, Faculty of Medicine (2015–106 and 2019–037). All patients and persons involved in this study gave written informed consent. The study was performed according to the rules of the declaration of Helsinki.

### Primary cell culture

Stellate cells were isolated from the tissues by using of the outgrowth method according to Bachem et al.^8^. Briefly, the tissue was cut directly after retrieval under sterile conditions into pieces of 3 mm and transferred to culture medium consisting of 400 ml low glucose Dulbecco’s Modified Eagle’s Medium (DMEM) (1000 mg/L, Sigma-Aldrich, Germany), 400 ml Ham ´s F12 Medium (Sigma-Aldrich, Germany), 160 ml fetal bovine serum (FBS) (Sigma-Aldrich, Germany), 10 ml penicillin/streptomycin (10.000 Units penicillin and 10 mg streptomycin per ml in 0.9% NaCl, Thermofisher, Germany) and 10 ml amphotericin B (250 μg / ml, Sigma-Aldrich, Germany). The tissues with 5 ml medium were cultured in the incubator at 37 ºC and 5% CO_2_. Every second day the medium was changed with 5 ml fresh one. The average culture time was three to four weeks until there were enough PSCs for further experiments.

### Immunostaining

Immunocytochemistry (ICC) on PSCs was performed with antibodies specific for periostin, GFAP, SERPINB2 and α-smooth muscle actin (Table 1). Briefly, 2.5 × 10^4^ of PSCs were seeded on the slides and incubated overnight, followed by fixation with − 20 °C cold Acetone (Honeywell, Germany) and air-drying on the next day. Thereafter, the cells were incubated at 4 °C for 20 min in a 4:1 solution of Methanol (Sigma, Germany) and H_2_O_2_, respectively. Slides were then incubated with the specific antibodies overnight in a wet chamber and washed with (Table 1) 1 × PBS (AppliChem, Germany). As a negative control, cells (without primary antibody) were incubated with PBS and Dako mouse IgG solution (Dako, Denmark) only. On the next day, the slides were incubated with an avidin–biotin-peroxidase complex (Dako, Denmark) according to manufacturer´s instructions. After 3 × 10 min washing in PBS, specific immunostaining was visualized with diaminobenzidine (DAB) chromogenic solution (1:50). Finally, cells were lightly counterstained with Mayer’s hematoxylin and photographed under light microscope.

**Table 1:** List of the used antibodies.

Primary antibodies	Origin	Cone	Company
α – smooth muscle actin	Mouse-monoclonal antibody Clon 1A4	8 μg/ml	Dako Denmark
Periostin	Rabbit-polyclonal antibody	2 μg/ml	Biovendor Germany
SERPINB2	Rabbit-polyclonal antibody	0,39 mg/ml	Thermo Fisher Scientific, USA
GFAP	Rabbit-polyclonal antibody	1:1000 (IF)	Abcam UK
Second antibody LSAB-Kit	Biotinylated anti-rabbit and anti-mouse immunoglobulin		Dako Germany
Alexa Fluor488	Goat anti Rabbit IgG H&L	2 μg/mL stock	Abcam UK
Hoechst	Nucleus coulor 33,342	10 mg/ml stock	Sigma-Aldrich Germany

### Immunofluorescence staining

To perform immunofluorescence staining, anti-glial fibrillary acidic protein (GFAP) (Abcam, UK) was used (Table 1). Slides were washed with 1 × PBS, fixed with 4% PFA and blocked with 3% bovine serum albumin (BSA) (Sigma, Germany) for 1 h. Anti-GFAP was diluted 1:1000 in Dako diluent (Dako, Denmark) and then applied overnight in a wet chamber at 4ºC. On the next day, the slides were washed with PBS. Following, Alexa Fuor488 (Abcam, UK) 1:400 diluted with PBS, was applied as a secondary antibody and incubated in a wet chamber in the dark for 1 h. Nucleus staining was performed with Hoechst 33,342 (Sigma, Germany) 1:100 in PBS, for 2 min in a wet chamber with cover. The Slides were repeatedly washed with PBS and distilled water and were inundated with Mounting Medium (Dako, Denmark) for microscopy.

### Oil red O staining

The Oil Red O Stock Solution (Sigma, Germany) was prepared by dissolving 0,5 g with 100 ml isopropanol protected from light. Cells were washed with 1 × PBS (AppliChem, Germany) and fixed with 4% PFA (Merck, Germany) for 15 min. Slides were covered with cells and 1 × PBS and sealed with parafilm for storage at 4 °C. Next, the slides were covered with Oil Red O working solution for 11 min. The OIL Red O solution was aspirated, the slides were washed afterwards and then covered with haematoxylin (Merck, Germany) for 10–30 s. After another washing step with 1 × PBS, the slides were rinsed with distilled water and covered with Aquatex (Merck, Germany).

### DNA isolation

DNA from PSCs and patient´s whole blood was isolated with QIAamp DNA Blood Mini Kit (Qiagen, Germany) according to manufacturer’s instructions.

### Next generation sequencing (NGS)

NGS analyses were performed with 5 pancreatic cancer samples at the Core facility of the University of Leipzig, Germany. 50 ng of genomic DNA were used for paired-end libraries synthesis with the Nextera DNA Library Prep kit (Illumina) according to the instructions of the manufacturer. A pool of up to 8 libraries was used for exome enrichment and indexing with the Nextera Rapid Capture Expanded Exomes kit (Illumina). Cluster generation was performed with the library pool at a concentration of 10 nM using an Illumina cBot. Paired-end reads of 2 × 150 bp were sequenced with an IlluminaHighScan-SQ sequencer at the sequencing core facility of the Faculty of Medicine (University Leipzig) using version 3 chemistry and flowcell according to the instructions of the manufacturer. After base calling with Real-Time Analysis software 1.13 (Illumina) demultiplexing of raw reads, adapter trimming and quality filtering was done according to Stokowy et al.^9^. Resulting read pairs were mapped to the human genome (hg38) using the BWA aligner^10^. Mapped reads were further processed for variant calling according to the Best Practices Workflow^11,12^, suggested by the Broad Institute using Genome Analysis Toolkit (GATK, version 3.4)^11^. Somatic variant detection was done using the program Mutect2 from GATK (Version 4.0.4.0) against a panel of normals consisting of the corresponding blood samples^13^. Finally, all variants were annotated using ANNOVAR^14^.

For bioinformatic analysis, the Software MuTect2 was used. Only the variants with a coverage over 30 were selected. Furthermore, four pathogenicity prediction scores SIFT, Polyphen2, CADD and the MutationTaster Score were used to focus on interesting candidates (https://sift.bii.a-star.edu.sg/), (https://cadd.gs.washington.edu/), (http://genetics.bwh.harvard.edu/pph2/dokuwiki/start), (http://www.mutationtaster.org/info/documentation.html). Variants were visualized with the Integrative Genomics Viewer (IGV) (https://software.broadinstitute.org/software/igv/). Only the variants with allele frequencies around 25% were taken into consideration and further analysed with Sanger Sequencing.

### PCR and Sanger sequencing

Amplification of the selected genes was performed with the primers presented in the Table 2 and Go Taq® polymerase according to the following conditions: hold 2 min at 95 °C, followed by 40 cycles of 30 s at 95 °C, 30 s at 60 °C and 30 s at 72 °C. Additional extension step at 72 °C for 30 s was included (Promega, Germany). The PCR was performed with a thermocycler (TRIO, Biometra). Products of PCR were separated on a 1% agarose gel and visualized with SYBR Safe (Thermo Fisher, Germany), followed by detection with ChemiDocTM Touch Gel Imaging System (Biorad, Germany).

**Table 2:** List of the used primer and their sequences.

Primer		Amplificate size	Sequence
SerpinB2	s	617 bp	CAC CTG CCT TCC ATA GCC AA ACT
	as		AAG CTC AGG GTC AAG CC
CNTNAP4	s	683 bp	ACT GGT CAG AAG CTG GAC TAC A TTG CTC
	as		TTC TTA ATT GCA GTG TGAT
DENND4B	s	530 bp	TGA GGT TTA TGC AGG CTG GG GAG
	as		AAC CAG TGA ACA GGG GG
DPP4	s	451 bp	AGA CCT TCA AAG TAA AGC CCA CTAA GGA
	as		TTA CCT CTT CTC AGT GCCA
FGFBP2	s	653 bp	ATT CCT GCA CTA TGC GTC CC
	as		AGT GCT AAG TGC CTC TCA CG
MIGA2	s	648 bp	ATC AGG CAG III GTG GGT CC
	as		TCC TCA AAC AGC TCC ATG CC
POLE	s	688 bp	GCC AGG TAA ATC GGG TCC TT
	as		GTG CGT GGT GGT ACA GGT AG
SNRNP40	s	533 bp	CCA CCA ATC CTG GTA TCG CA
	as		GGA ACA CGT ACG CAG CAT TC
TOP2B	s	662 bp	AGA AGA CAA AAG GAC AAC ACA AGA
	as		TCC ACC TCG GTG ATG CTT T
ZDHHC18	s	482 bp	TGC CCA AGG CTC M! GTG AT
	as		GCA TTA TCC CAG CAG TCC GT

S = sense; AS = antisense.

The Sanger Sequencing was performed in the SeqLab (Göttingen, Germany) with a Barcode economy run. The samples were purified with the Exo- AP method (25 μl Exonuclease, 5 Unit, New England Bio Labs, United Kingdom and 25 μl Antarctic Phosphatase, 1 Unit, New England BioLabs, United Kingdom). For the visualization and verification of the mutations, FinchTV (https://finchtv.software.informer.com/1.4/) and BLAST (https://blast.ncbi.nlm.nih.gov/Blast.cgi) software were applied, respectively.

### Functional analysis

For a further investigation of the functional role of the targeted point mutation in the *SERPINB2* gene we performed a functional analysis using immortalized fibroblasts (SV-80, CLS Cell Lines Service GmbH, Eppelheim, Germany).

### Transfection

A PAI-2 (*SERPINB2)* CRISPR/Cas9 KO plasmid (Santa Cruz, US) was used to create the knockout model (a knockout strategy was used and not a CRISPR-engineered mutation mimicking the identified premature stop codon). The day before transfection of the KO and the control plasmid, 1 × 10^5^ cells per well were seeded into a 24-well plate. 1 µg of plasmid-DNA was carefully dissolved and mixed with 50 µl of Opti-MEM I Reduced Serum Medium (Thermo Fisher Scientific, Germany). After dissolving Lipofectamin 2000 (Thermo Fisher Scientific, Germany) in Opti-MEM for 5 min at room temperature, the solution was mixed with the dissolved plasmid-DNA. Following 20 min of incubation 100 µl of the final solution were transferred into one well of a 24-well plate and incubated for 24 h (at 37Cº and 5%CO_2_). Next, 0.15 µg/ml Puromycin were used for selection. Cell colonies of clones were selected under light microscopy.

### RNA Isolation and qPCR

RNA was isolated according to manufacturer’s protocol using direct-zol™ Miniprep Kit (ZYMO RESEARCH EUROPE GMBH, Germany). For quantitative analysis, we performed qPCRs using the QuantStudio3 system (Thermo Fisher Scientific, Germany). For cDNA preparation the High-Capacity cDNA Reverse Transcription Kit (Thermo Fisher Scientific, Germany) was applied. RT-qPCR was performed using HOT FIREPol® EvaGreen® qPCR Mix Plus (ROX) (Solis BioDyne, Estonia). Primers were obtained from Thermo Fisher Scientific, Germany. Sequences are available upon request.

### Western blot

To analyse the knock down of *SERPINB2* expression on the protein level, western blot was performed in addition to ICC staining. Proteins were extracted according to standard procedures from cultured cells^15^. Equal amounts of protein were loaded and separated by SDS-PAGE using a 12% SDS acrylamide gel. After transfer, PVDF Blotting Membranes (GE Healtcare Life science, Great Britain) were incubated with SerpinB2 antibody (1:500) (Thermo Fisher Scientific, Germany) and monoclonal rabbit antibody (1:10.000). α-tubulin (Thermo Fisher Scientific, Germany) was used as loading control (1:500). Bands were detected by chemiluminescence and quantified with densitometry.

### Proliferation assay

Cells were seeded in 24-well plates (25 × 10^3^ per well) in PSC-growth-medium as described above. After 24, 48 and 72 h of incubation at 37 °C in 5% CO2, Thiazolyl Blue Tetrazolium Bromide (dissolved in HBSS, Sigma- Aldrich, US) was added to the wells. At each time point DMSO (Merck, Germany) was added after another four hours of incubation. Absorbance was measured at 570 nm after the well plate was vigorously shaken.

### Wound healing/migration assay

Cells were seeded in 12-well plates (300.000 cells per well) in PSCs growth medium, which was replaced by serum reduced medium (0,1% FCS) the next day. After medium-starving the cells overnight, a wound was scratched through the cell monolayer in each well (with a 100 µl pipette tip) the next morning. For quantification of wound closure, pictures were taken at timepoint zero, after four and after eight hours.

### Contraction assay

Collagen gel contraction assay was performed using a Cell Contraction Assay kit (Cell Biolabs, US). Briefly, Collagen Gel Working Solution was prepared on ice according to the manufacturer’s protocol and was added to a 2 × 10^6^ cells/ml cellsuspension in a 4:1 ratio. 500 µl of the cell-collagen mixture was added per well in a 24-well plate and incubated for polymerization for one hour at 37 °C as well as 1 ml of PSCs growth medium. After two days of incubation the collagen gels were released from the sides of the culture dishes. Pictures were taken 1, 3 and 5 days after releasing the gels to quantify the change in size of the collagen gels.

## Results

**Figure 1:**
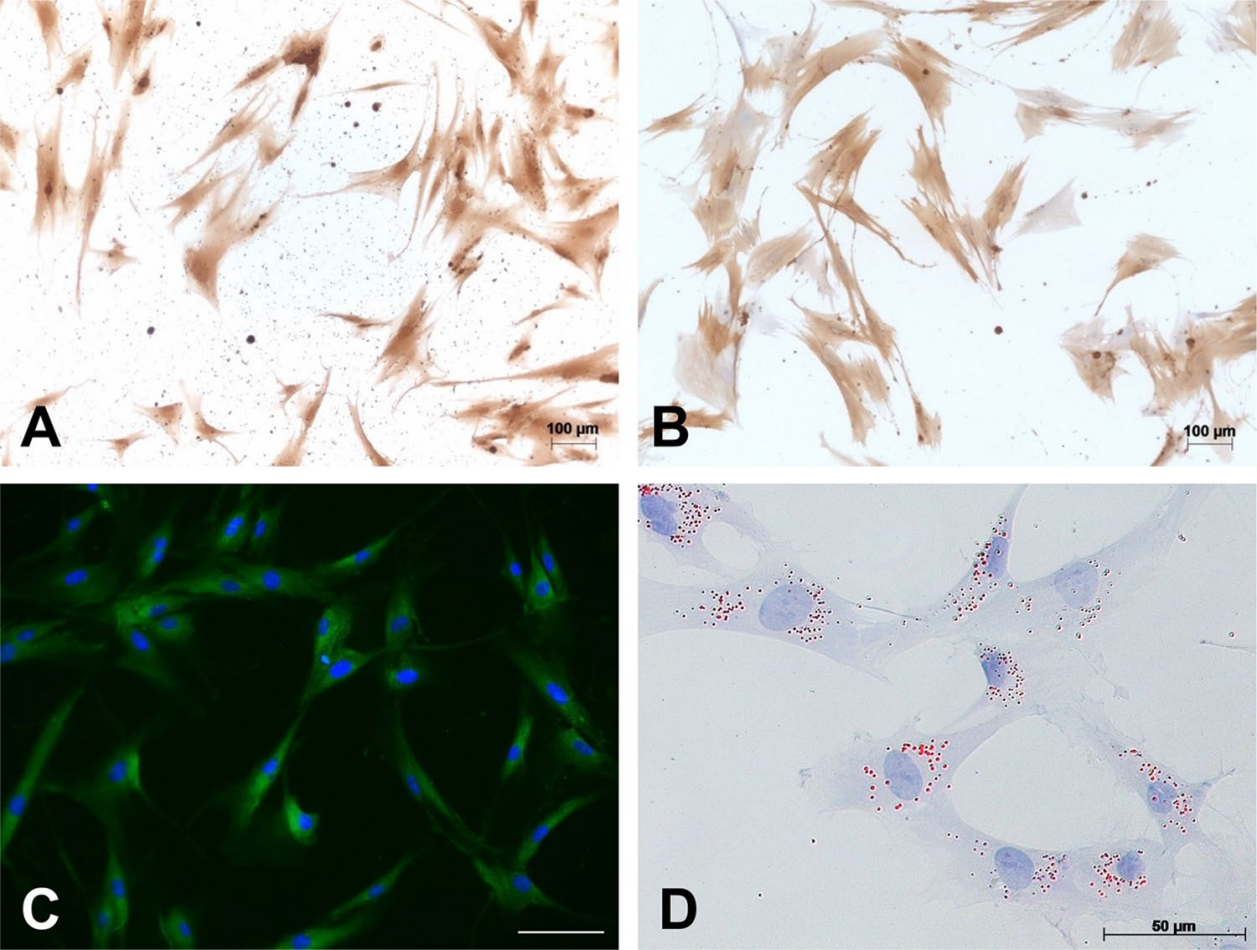
Immunocytochemistry (ICC) of PSCs with Periostin, α—Smooth Muscle Actin, Immunofluorescence (IF) of GFAP and Oil Red staining. Cells display strong cytoplasmatic staining with Periostin (**A**), α-SMA (**B**) and GFAP (**C**). Oil red staining of fresh isolated PSCs after 7 days of culture visualize cytoplasmatic vitamin A-containing lipid droplets (**D**). Magnification: 40×.

Pancreatic stellate cells (PSC) were isolated by the outgrowth method from patients operated on for pancreatic ductal adenocarcinoma or chronic pancreatitis. Immunocytochemistry for periostin, α-smooth muscle actin and immunofluorescence for GFAP as well as OilRed staining confirmed that the cells were PSCs (Fig. 1). DNA was extracted from PSC (*n* = 50 patients) as well as from blood samples of the same patients.

### NGS analyses

**Figure 2:**
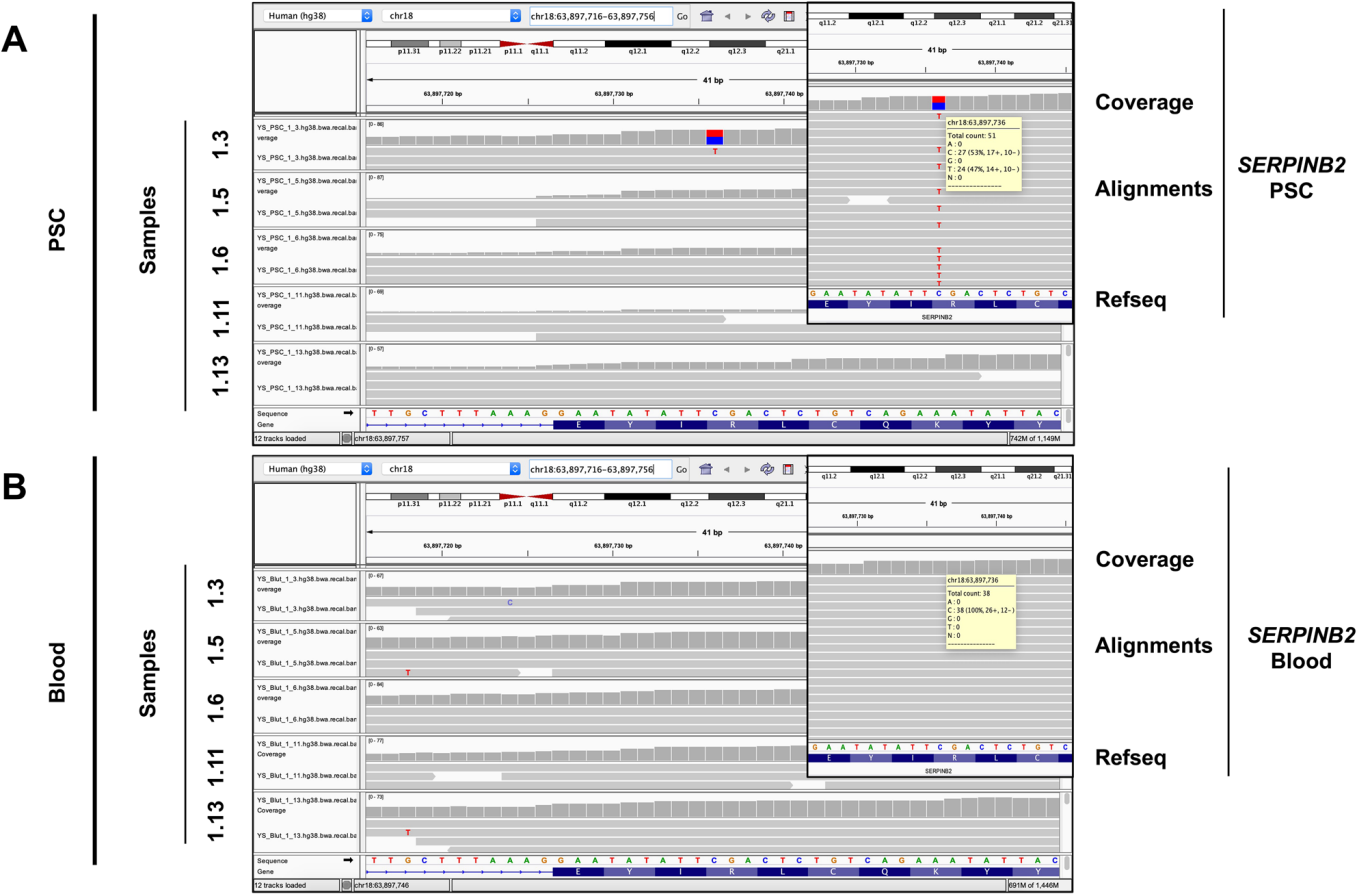
PSCs show unique point mutations in their genomes compared to matching blood counterparts at selected gene locations. Visualization of selected point mutation of the SERPINB2 gene in PSC samples with the IGV tool. Direct comparison to base sequences at the same gene localization of matched blood samples show no variant verifying the change in the PSCs genome as a somatic mutation.

Initially, NGS analysis (*n* = 5 patients) showed between 2,559 to 5,816 variants per sample (PSC versus blood DNA). To select somatic point mutations, the program GATK was used and a coverage of 9 and an allele frequency of 0.1 was set as a cut-off. This analysis revealed on average 26 variants per sample (range 7–76) in the DNA of PSCs compared to the paired blood samples (suppl. Tables 1–5). All variants were confirmed manually utilizing the IGV viewer (an example is shown in Fig. 2). Based on a coverage of at least 30 and an allele frequency of at least 25%, 10 genes were chosen for further analysis: *SERPINB2*, *CNTNAP4*, *DENND4B*, *DPP4*, *FGFBP2*, *MIGA2*, *POLE*, *SNRNP40*, *TOP2B* and *ZDHHC18* (Table 3).

**Table 3:** List of identified genes.

Gene name	Chr	Start	End	Ref	Alt	AD	Func. ref Gen	Exonic func
*SERPINB2*	chr18	63.897.736	63.897.736	C	T	30	exonic	stopgain
*MIGA2*	chr9	129.049.390	129.049.390	G	A	90	exonic	nonsynonymous SNV
*TOP2B*	chr3	25.598.380 25.598.384	25.598.380 25.598.384	G G	C A	79 82	exonic exonic	nonsynonymous SNV
*ZDHHC*	chrl	26.851.209	26.851.209	C	G	106	exonic	nonsynonymous SNV
*CNTNAP4*	chr16	76.448.059	76.448.059	T	A	27	exonic	nonsynonymous SNV
*DENND4B*	chrl	153.940.945	153.940.945	G	A	99	exonic	nonsynonymous SNV
*DPP4*	chr2	162.047.459	162.047.459	G	T	73	exonic	nonsynonymous SNV
*FGFBP2*	chr4	15.962.573	15.962.573	G	C	64	exonic	nonsynonymous SNV
*POLE*	chr12	132.643.477	132.643.477	C	T	10 1	exonic	stopgain
*SNRNP40*	chrl	31.296.714	31.296.714	G	A		exonic	nonsynonymous SNV

Data of the somatic point mutations in correlation to localization, base exchange, and function. SNV: single nucleotide variant. Chr.: chromosome. Ref.: reference. Alt.: alternative. AD: coverage.

### Data of the mutations of interest

The variants of greatest interest according to the MuTect2 analysis data are presented in Table 3. Generally, GATK identified somatic point mutations. Genomic coordinates were visualized using the human genome 38 (hg38) as a reference sequence. All mutations were exonic. Mutations in *SERPINB2* and *POLE* were predicted as stop gains. Further mutations are nonsynonymous single nucleotide variants, meaning the mutations caused a change in the amino acid sequence. In the majority of cases, the reference bases were Guanine (*n* = 7) or Cytosine (*n* = 3). The alternative bases were mostly Adenine (*n* = 5) and Thymine (*n* = 3). The coverage of the mutations (AD) had a range from 29 to 106.

### Pathogenicity prediction

Next, pathogenicity prediction scores were analyzed for the selected variants (Table 4). The scores were used to select interesting and relevant mutations. For the *SERPINB2* gene, there were no SIFT and Polyphen2 data. Nevertheless, a *CADD* Score of 35, which suggested the mutation might be pathogenic. The cutoff for the highest pathogenicity prediction call is 30. The MutationTaster had a maximum value of 1 such as in all cases except for the *MIGA2* gene (0.9). The mutation in the *MIGA2* gene showed a SIFT score of 0.075. The cutoff value for this score for being pathogenic is under 0.05. Referring only to this score, the mutation would not be predicted as pathogenic. But a value of 0.999 in the Polyphen2 Score is very close to the maximum of 1, that predicted this mutation to be very pathogenic. The CADD Score value was 23.6 for this mutation which also predicted pathogenicity. The first mutation in the *TOP2B* gene had a SIFT score of 0.009 and the second one a SIFT Score of 0, which were predicted to be pathogenic. The Polyphen2 Scores of both mutations were with values of 0.978 and 1 very close to and the maximum. Moreover, the CADD scores were 25.4 and 28.6, which predicted pathogenicity as well. The mutations in the *ZDHHC* and *CNTNAP4* gene had SIFT scores of 0.006 and 0.007, close to the maximum of 0. Polyphen2 scores were at maximum values of 1 and close to the maximum with 0.84. The CADD Scores were 27.8 and 24.8. The mutation in the *DENND4B* gene reached the maximum value in every score (SIFT = 0; Polyphen2 = 1, CADD = 34). In contrast the mutation in the *DPP4* gene had only values of 0.3 in SIFT, 0 in Polyphen2 and 9.7 in CADD. For the mutation in the *POLE* gene were only data in the CADD Score found (CADD = 41). Finally, the last mutations in the *FGFBP2* and *SNRNP40* gene had SIFT scores of 0.061 and 0.028, Polyphen2 scores of 0.99 and 0.695 and CADD scores of 16.3 and 23.7.

**Table 4:** Overview about pathogenicity prediction scores and the allele frequencies (AF).

Gene name	Score				
	SIFT	Polyphen2	CADD	MutationTaster	AF (%)
*SERPINB2*			35	1	43
*MIGA2*	0.075	0.999	23.6	0.9	41
*T0P2B*	0.009 0	0.978 1	25.4 28.6	^1^	35 35
*ZDHHC*	0.006	1	27.8	1	34
*CNTNAP4*	0.007	0.84	24.8	1	32.8
*DENND4B*	0	1	34	1	46.3
*DPP4*	0.3	0	9.7	1	28.1
*FGFBP2*	0.061	0.99	16.3	1	37.5
*POLE*			41	1	31.4
*SNRNP40*	0.028	0.695	23.7	1	29.5

CADD: Combined Annotation Dependent Depletion.

The scores were used to select interesting and relevant mutations. SIFT: Sorting Intolerant From Tolerant.

### Sanger sequencing

To confirm the detected variants of the ten selected genes in PSCs compared to blood DNA, Sanger sequencing was carried out in the original five cases and in additional samples of DNA isolated from PSCs of 45 patients. Nine of ten mutations could be reproduced by Sanger sequencing. However, none of the variants were detected in the additional 45 cases.

### Functional analysis

**Figure 3:**
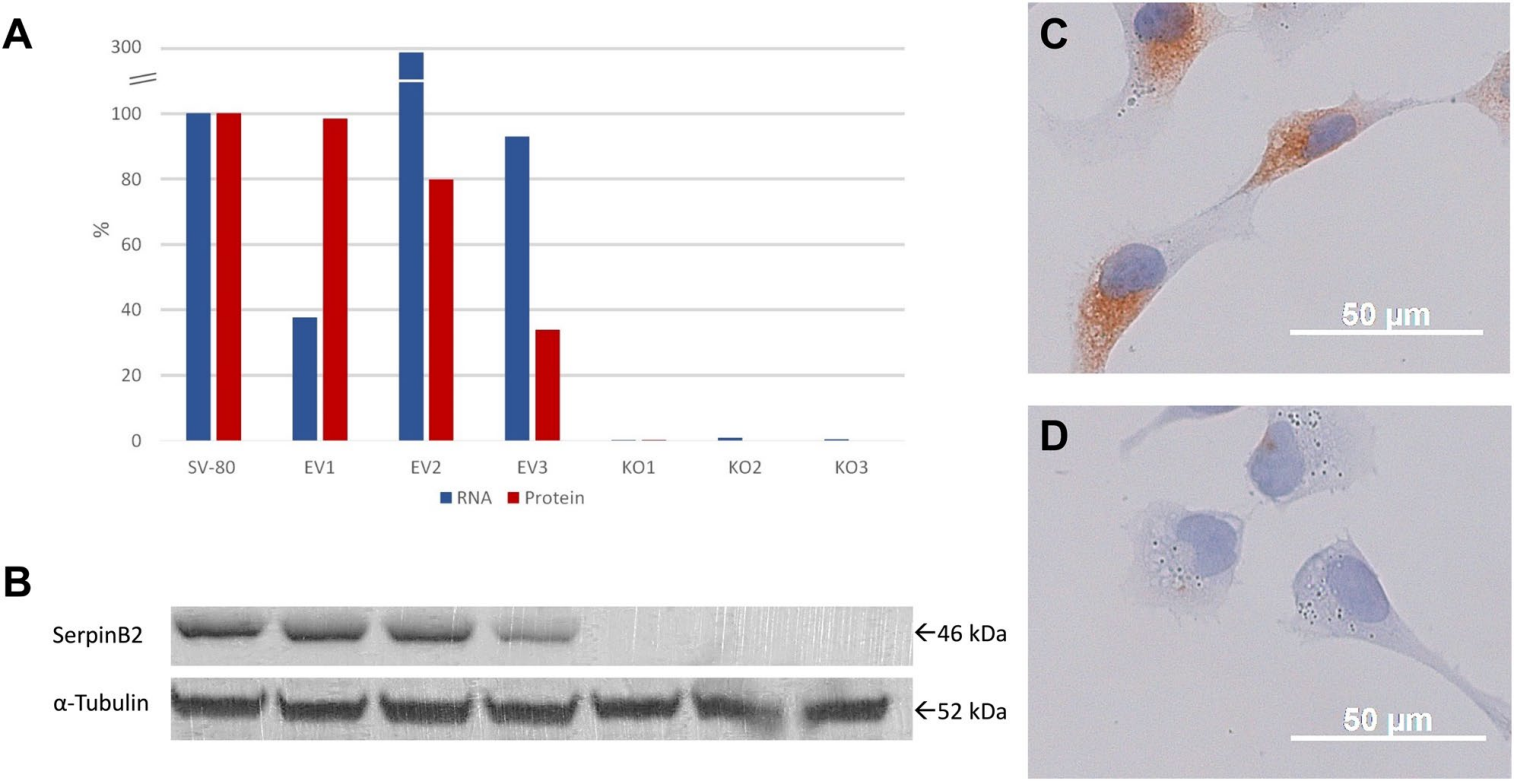
Knock out of *SERPINB2* in fibroblasts: qPCRs demonstrating *SERPINB2* mRNA levels at or below the level of detection in the analyzed clones KO1-3, but not in the control clones EV1-3 (**A**). Western blot analysis revealed knock-out on the protein level for KO1-3 as well (**B**). Densitometry analysis is shown in A. Immunocytochemistry for *SERPINB2* demonstrating strong staining in the control clones (**C**) and reduced to absent staining in knock-out clones (**D**).

The mutation of the *SERPINB2* gene was investigated for functional relevance. To this end, immortalized fibroblasts were transfected with a SERPINB2 CRISPR/Cas9 KO plasmid. qPCR confirmed that the *SERPINB2* gene was knocked out in the analyzed clones, but not in the empty vector control clones (Fig. 3A). Western blot analysis revealed knock out on the protein level as well (Fig. 3A, B), which was also confirmed by immunocytochemistry (Fig. 3C, D).

**Figure 4:**
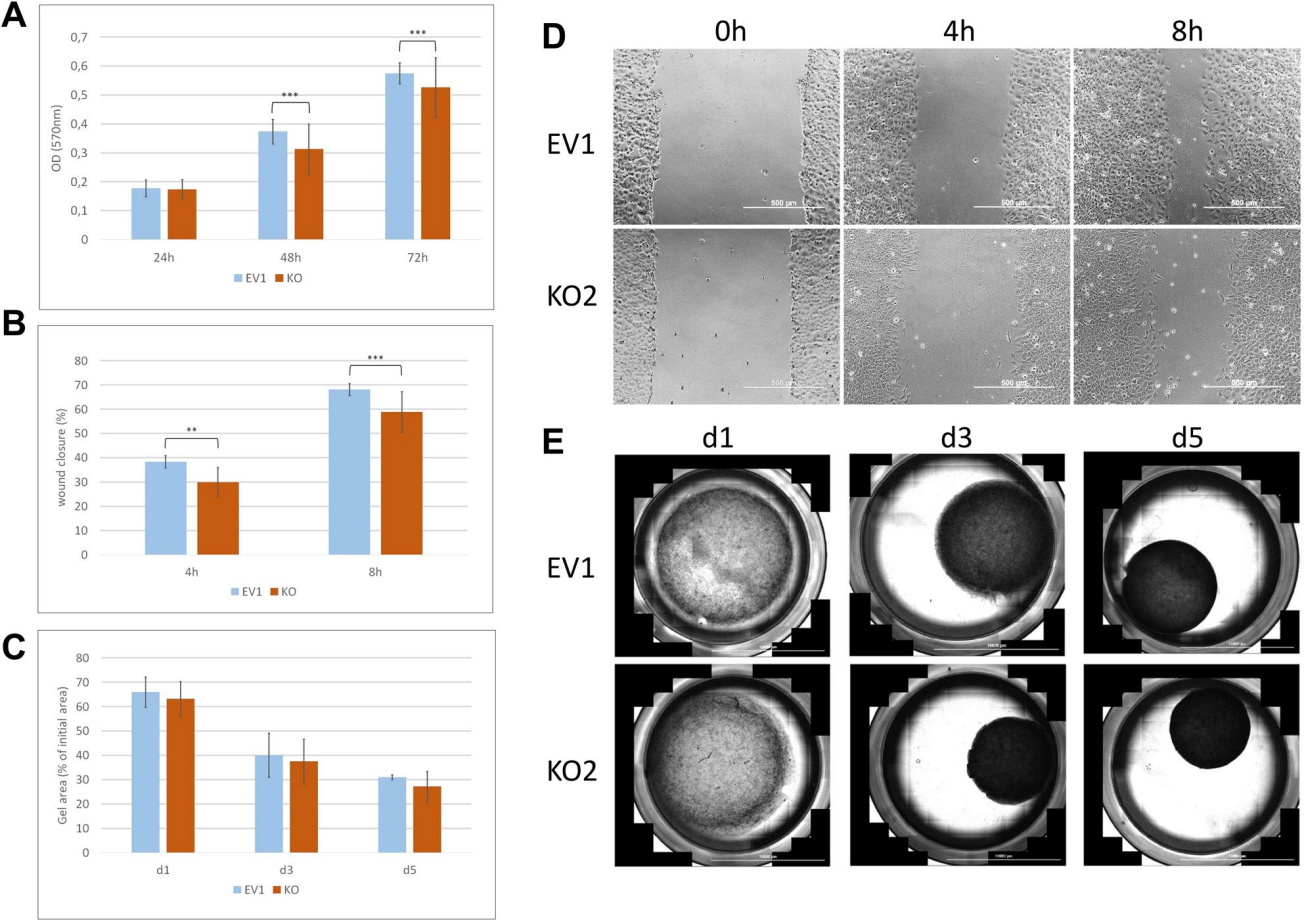
Functional analysis: (**A**) Proliferation assays of SERPINB2 knock-out (KO) and control (EV1) clones. (**B**) Scratch assays of SERPINB2 knock-out (KO) and control (EV1) clones. An example is shown in D. (**C**) Contraction assays of SERPINB2 knock-out (KO) and control (EV1) clones. An example is shown in E. Data are pooled from three experiments of the EV1 control clone and three knock-out clones. **p < 0.01; ***p < 0.001.

Proliferation assays (Fig. 4A) revealed no relevant differences in growth after 24 h for KO clones versus control clones. In contrast, after 48 h and 72 h, knock-out clones displayed slightly reduced growth as compared to the control clones (*p* < 0.001). Similarly, scratch assays (Fig. 4B) demonstrated significantly reduced migration in *SERPINB2*-knock-out clones as compared to the control clones at 4 h and 8 h (an example is shown in Fig. 4D). Contraction assays (Fig. 4C) did not reveal a significant difference between knock-out and control clones at any time point (an example is shown in Fig. 4E).

## Discussion

Pancreatic cancer is characterized by a generally poor prognosis with an overall 5-year survival rate of 10%^16^. With its main risk factors such as age, tobacco smoking, obesity, and low physical activity, it is projected to become the second leading cause of cancer-related deaths within this decade^17^. On the molecular level, key driver mutations have been identified. The *KRAS* gene is altered by activating mutations, and the *CDKN2A* gene by inactivating mutations or other mechanisms (both in > 90% of tumors)^1^. Furthermore, inactivating mutations in the *TP53* gene (around 70%) and *SMAD4* gene (close to 60%) are enriched in this aggressive tumor type. A large number of other mutations, including *ARID1A*, *GL13*, *MLL3* and *DNAH11*, are altered less often (in around 10% of cases)^1^, but might have functional relevance in individual cases.

Further, pancreatic tumors exhibit the characteristic of an extremely dense stroma constituting around 90% of the tumor mass^18^. The stroma plays an important role in the tumor biology by creating hypoxic conditions, acting as a potential mechanical barrier of tumor cell spread, but also diminishing efficacy of utilized chemotherapies. They further impact on carcinogenesis by increasing matrix stiffness in the tumor microenvironment directly activating epithelial-mesenchymal transition (EMT), tumor invasion, and metastasis of tumor cells^19^. Pancreatic stellate cells constitute the majority of cancer-associated fibroblasts (CAFs) in pancreatic cancer and play a crucial role in inducing this desmoplastic reaction trough production of huge amounts of ECM proteins^20^. Their role in the microenvironment of pancreatic cancer is complex with both tumor-promoting and tumor-restraining functions being described and observed in vitro and in vivo.

It is generally assumed that genetic alterations drive neoplastic transformation in primary tumor cells and that stromal cells react on these altered cells. It is not known whether PSCs acquire genetic alterations with functional relevance during pancreatic carcinogenesis. In the present study, we therefore analyzed cancer derived PSC versus matched blood genome in an exploratory cohort (*n* = 5) and confirmed findings in a larger cohort (*n* = 50). The absence of key pancreatic cancer mutations *KRAS*, *P53*, *P16* and *SMAD4* ruled out contamination with tumor cells. Importantly it also reveals that PSCs do not harbor these cardinal mutations associated with pancreatic cancer cells. Ten potential pathogenic mutations (in the *SERPINB2*-, *CNTNAP4-*, *DENND4B-, DPP4-*, *FGFBP2-*, *MIGA2-, POLE-*, *SNRNP40-*, *TOP2B-* and *ZDHHC18* genes) could be identified and confirmed in individual cases, yet no mutation was observed in more than one of the 50 analysed cases (including also chronic pancreatitis). It is therefore unlikely that common driver mutations exist in PSCs in pancreatic cancer.

From our data we cannot answer the question whether all CAFs in one tumor share mutations, or whether there are many clones present in the tumor mass. However, our lower limit of sequence coverage (30x), a variant allele frequency (VAF) of about 25% is based on the detection of at least seven mutated alleles. So, there is rather little room in the source data that would hide other somatic mutations for the following reasons. 1) detection of less than seven mutated alleles would gradually increase the chance to make a false positive mutation call. 2) of the few variants that fail our threshold, none of them would suggest a functional contribution as judged from the prediction of their pathogenicity. Besides avoiding false-positive mutation calls, a VAF above 25% also underscores the functional contribution of the respective variant. Because our method to outgrow the CAF avoids contamination by non-CAF cells, about half of the outgrowing cells would carry this variant allele if the VAF is 25%. So, it is more likely that our cut off helps to detect major subclones of the original CAF clone in the tumour. However, it does not exclude that there are smaller minor clones, but their detection is beyond the limits of our methodology.

The functional relevance of the observed mutations in PSCs in the microenvironment of pancreatic cancer is currently not known and is the focus of future studies. Interestingly, however, the detected *SERPINB2* mutation is predicted to result in a stop and therefore the expression of a functional *SERPINB2* protein would be lost in those PSCs in pancreatic cancer. A premature stop might have different effects as compared to a knockout, but both result in a non-functional protein.

A study by Harris et al. analyzed the role of *SERPINB2* in stromal remodeling and local invasion in pancreatic cancer^21^. There, *SERPINB2 *^*-/-*^ mouse embryonic fibroblasts (MEFs) were compared to wild-type MEFs. It was demonstrated that wild-type MEFs were highly mobile, moving freely while *SERPINB2 *^*-/-*^ MEFs were widely immobile. Phenotypically, *SERPINB2 *^*-/-*^ MEFs had fewer but longer and more stable protrusions in contrast to wild-type MEFs that had numerous and short protrusions. Allograft experiments with nude mice (PDAC cells and MEFs (wild-type or *SERPINB2 *^*-/-*^) were co-injected) displayed PDAC tumors to be larger in *SERPINB2 *^*-/-*^ MEFs than in wild-type MEFs. It was concluded that SERPINB2 plays a major role in the inhibition in PDAC local invasion trough regulation of stromal collagen remodeling. In line with these observations, we could show that SERPINB2 knock-out resulted in reduced growth and migration, yet not collagen contraction. Another study by Westrick et al. investigated that a deficiency SERPINB2 results in accelerated tumor growth^22^, which was not due to a specific deficiency within macrophages or other hematopoietic-derived cell population^22^, suggesting that *SERPINB2* has a regulatory function in non-tumor cell types as endothelial cells and fibroblasts^22^.

Taken together, it is conceivable that the identified *SERPINB2* mutation in cancer-associated PSCs results in enhanced pro-tumorigenic activity of PSCs.

While specific mutations (such as *SERPINB2)* might contribute to tumor biology in individual cases, it is also conceivable that most of the identified mutations represent passenger mutations that do not have functional effects in pancreatic cancer-associated PSCs.

Typically, alterations develop due to several risk factors such as aging, mutagenic chemicals, radiation, ultraviolet (UV) light, oxygen radicals and further factors^23^. Many alterations are defined as passengers with the ability to remain lifelong in normal tissues without having functional consequences or causing tumor development. A subdivision of genetic and epigenetic alterations composes genetic alterations as a result of aging, mutagenic chemicals, radiation and UV-light while epigenetic alterations occur next to aging through chronic inflammation, cigarette smoking and others^23^. Somatic mosaicism is a phenomenon derived from the presence of multiple cell clones containing distinct genotypes in the same individual^24^. For instance, endogenous factors like DNA-double-strand break, inefficient DNA repair, DNA polymerase slippage and mobile elements lead to somatic mutations. Just like exogenous factors such as nicotine and UV exposure. Furthermore, there is a de novo locus-specific rate enabling genomic alterations (10^–6^ to 10^–4^) per locus and generation^25,26^. Further analysis, including single-cell analysis and comparison to other cell types would be necessary to investigate accumulations of somatic mutations and their heterogenic functions in cancer-associated human PSCs.

In conclusion, the existence of common driver like mutations in PSCs is unlikely. Nonetheless, the identified individual mutations in tumor-associated PSC genomes might affect PSCs themselves and the microenvironment of tumors. Further studies with larger number of patients, additional control tissues/cells (e.g. healthy pancreatic tissue), as well as functional studies of individual mutations are necessary. An in-depth knowledge of individual alterations -not only of cancer cells but also of non-neoplastic tumor associated cells- provides new options for investigations to generate better therapies against pancreatic cancer in the future.

## Supplementary Information

Supplementary Information.

## Data Availability

All data generated or analyzed during this study will be provided by corresponding author at reasonable request.

## References

[1] Kleeff J (2016). Pancreatic cancer. Nat. Rev. Dis. Primers.

[2] Rosato V (2015). Population attributable risk for pancreatic cancer in Northern Italy. Pancreas.

[3] Xue R (2018). A rising star in pancreatic diseases: Pancreatic stellate cells. Front. Physiol..

[4] Sahai E (2020). A framework for advancing our understanding of cancer-associated fibroblasts. Nat. Rev. Cancer.

[5] Apte MV, Wilson JS, Lugea A, Pandol SJ (2013). A starring role for stellate cells in the pancreatic cancer microenvironment. Gastroenterology.

[6] Vonlaufen A (2008). Pancreatic stellate cells: Partners in crime with pancreatic cancer cells. Cancer Res..

[7] Erkan M (2010). Organ-, inflammation- and cancer specific transcriptional fingerprints of pancreatic and hepatic stellate cells. Mol. Cancer.

[8] Bachem MG (1998). Identification, culture, and characterization of pancreatic stellate cells in rats and humans. Gastroenterology.

[9] Stokowy T (2014). Analysis options for high-throughput sequencing in miRNA expression profiling. BMC Res. Notes.

[10] Li H, Durbin R (2010). Fast and accurate long-read alignment with Burrows-Wheeler transform. Bioinformatics.

[11] McKenna A (2010). The genome analysis toolkit: A MapReduce framework for analyzing next-generation DNA sequencing data. Genome Res.

[12] Van der Auwera GA (2013). From FastQ data to high confidence variant calls: the Genome Analysis Toolkit best practices pipeline. Curr. Protoc. Bioinf..

[13] Cibulskis K (2013). Sensitive detection of somatic point mutations in impure and heterogeneous cancer samples. Nat. Biotechnol..

[14] Wang K, Li M, Hakonarson H (2010). ANNOVAR: functional annotation of genetic variants from high-throughput sequencing data. Nucleic Acids Res.

[15] Erkan M (2005). Loss of BNIP3 expression is a late event in pancreatic cancer contributing to chemoresistance and worsened prognosis. Oncogene.

[16] Siegel RL, Miller KD, Fuchs HE, Jemal A (2021). Cancer statistics, 2021. CA Cancer J Clin.

[17] Rahib L (2014). Projecting cancer incidence and deaths to 2030: the unexpected burden of thyroid, liver, and pancreas cancers in the United States. Cancer Res..

[18] Erkan M (2012). StellaTUM: current consensus and discussion on pancreatic stellate cell research. Gut.

[19] Laklai H (2016). Genotype tunes pancreatic ductal adenocarcinoma tissue tension to induce matricellular fibrosis and tumor progression. Nat. Med..

[20] Erkan M (2009). Cancer-stellate cell interactions perpetuate the hypoxia-fibrosis cycle in pancreatic ductal adenocarcinoma. Neoplasia.

[21] Harris NLE (2017). SerpinB2 regulates stromal remodelling and local invasion in pancreatic cancer. Oncogene.

[22] Westrick RJ (2020). Deficiency of plasminogen activator inhibitor-2 results in accelerated tumor growth. J. Thromb. Haemost..

[23] Takeshima H, Ushijima T (2019). Accumulation of genetic and epigenetic alterations in normal cells and cancer risk. NPJ. Precis. Oncol..

[24] De S (2011). Somatic mosaicism in healthy human tissues. Trends Genet..

[25] Lupski JR (2007). Genomic rearrangements and sporadic disease. Nat. Genet..

[26] Itsara A (2010). De novo rates and selection of large copy number variation. Genome Res.

