# Supplementary material for: Analysis of genomic alterations in cancer associated human pancreatic stellate cells
Source: Sci Rep. 2022 Aug 8;12:13532. doi: 10.1038/s41598-022-17748-1 (PMC9360052; doi:10.1038/s41598-022-17748-1)
Supplement: Supplementary file 1 — Supplementary Information. [file 41598_2022_17748_MOESM1_ESM.pdf]

Suppl. Table 1

ChrZ	Start	End	Ref	Alt	AD	Gene.refGene
chr1	26851209	26851209	C	G	71	*ZDHHC18*
chr1	31296714	31296714	G	A	50	*SNRNP40*
chr1	32695527	32695527	C	G	32	*SYNC*
chr1	153940945	153940945	G	A	55	*DENND4B*
chr1	157770462	157770462	C	A	16	*FCRL2*
chr1	158355468	158355468	A	G	23	*CD1E*
chr1	175160807	175160807	C	-	50	*KIAA0040*
chr1	175160809	175160819	T	-	54	*KIAA0040*
chr1	200407081	200407081	C	A	48	*ZNF281*
chr1	248099860	248099860	C	A	35	*OR2L13*
chr1	248593569	248593569	G	T	33	*OR2T10*
chr2	131071870	131071870	G	C	20	*FAM168B*
chr2	162047459	162047459	G	T	66	*DPP4*
chr3	25598380	25598380	G	C	62	*TOP2B*
chr3	25598384	25598384	G	A	61	*TOP2B*
chr3	52519980	52519980	C	T	66	*STAB1*
chr3	57287942	57287942	G	T	23	*ASB14*
chr3	112991112	112991112	G	T	26	*GTPBP8*
chr3	124717323	124717323	C	T	41	*KALRN*
chr3	172924251	172924251	C	T	65	*SPATA16*
chr4	15962573	15962573	G	C	32	*FGFBP2*
chr5	61526388	61526388	A	-	44	*ZSWIM6*
chr5	147831523	147831523	C	A	41	*SPINK1*
chr5	177097588	177097588	C	T	73	*FGFR4*
chr6	5368598	5368598	G	A	30	*FARS2*
chr6	24145741	24145741	C	A	33	*NRSN1*
chr6	36211417	36211417	A	G	57	*BRPF3*
chr7	92042078	92042079	AG	-	70	*AKAP9*
chr7	101040366	101040366	G	A	63	*MUC17*
chr7	128758997	128759006	A	-	72	*CALU*
chr7	140687239	140687239	C	A	17	*ADCK2*
chr8	51423669	51423669	G	C	97	*PXDNL*
chr8	81694806	81694806	T	C	36	*SLC10A5*
chr9	129049390	129049390	G	A	49	*MIGA2*
chr10	26173938	26173938	G	T	57	*MYO3A*
chr10	47368027	47368027	G	T	68	*ZNF488*
chr10	101023593	101023593	C	A	66	*PDZD7*
chr10	128106848	128106849	TG	-	43	*MKI67*
chr11	1929813	1929813	A	G	58	*TNNT3*
chr11	26995113	26995113	G	T	72	*FIBIN*
chr11	46385808	46385808	C	T	10	*CHRM4*
chr11	58403321	58403321	G	A	74	*OR5B3*
chr11	92982029	92982029	G	T	56	*MTNR1B*
chr11	109423969	109423969	-	G	53	*C11orf87*
chr11	129914951	129914951	G	A	37	*PRDM10*
chr12	6601984	6601984	C	A	41	*CHD4*
chr12	56326712	56326712	G	T	85	*PAN2*
chr12	119523766	119523766	G	A	37	*CCDC60*
chr12	132643477	132643477	C	T	52	*POLE*
chr13	26681250	26681250	C	-	21	*WASF3*
chr13	32770442	32770442	A	-	45	*PDS5B*
chr13	77007548	77007548	A	-	38	*FBXL3*
chr13	114257211	114257211	G	T	56	*CDC16*
chr15	51212388	51212388	C	T	43	*CYP19A1*
chr15	74625839	74625839	G	A	17	*CLK3*
chr16	76448059	76448059	T	A	29	*CNTNAP4*
chr17	12739289	12739289	C	-	47	*MYOCD*
chr17	65536338	65536338	G	T	25	*AXIN2*
chr17	67854490	67854490	G	T	59	*BPTF*
chr17	80332214	80332214	G	T	13	*RNF213*
chr17	82029185	82029185	G	T	53	*LRRC45*
chr18	34794215	34794215	C	A	27	*DTNA*
chr18	63897736	63897736	C	T	23	*SERPINB2*
chr19	1008657	1008657	G	A	66	*GRIN3B*
chr19	3784863	3784863	C	A	63	*MATK*
chr19	8511689	8511689	G	T	14	*ZNF414*
chr19	10702186	10702186	G	A	116	*QTRT1*
chr19	11245592	11245592	G	A	60	*DOCK6*
chr19	19026045	19026045	G	T	27	*SUGP2*
chr19	44903065	44903065	C	A	20	*TOMM40*
chr19	53407927	53407927	G	T	87	*ZNF765*
chr20	35502597	35502597	G	T	46	*CEP250*
chr20	52153046	52153046	C	A	55	*ZFP64*
chr21	30215330	30215330	A	T	95	*CLDN8*
chrX	68192920	68192920	A	-	25	*OPHN1*
chrX	69163167	69163167	C	A	39	*PJA1*

Suppl. Table 2

**Chr**	**Start**	**End**	**Ref**	**Alt**	**AD**	**Gene.refGene**
**chr2**	210315042	210315042	T	-	26	*MYL1*
**chr2**	227259814	227259814	C	A	61	*COL4A3*
**chr4**	55470786	55470786	-	A	35	*CLOCK*
**chr4**	139045292	139045292	T	G	42	*NOCT*
**chr5**	179644924	179644924	G	A	33	*C5orf60*
**chr8**	100712766	100712766	-	A	58	*PABPC1*
**chr9**	14789074	14789074	C	A	24	*FREM1*
**chr10**	33330769	33330769	G	A	35	*NRP1*
**chr10**	110810413	110810413	C	T	63	*RBM20*
**chr11**	121157952	121157952	C	G	74	*TECTA*
**chr12**	54400859	54400859	G	T	26	*ITGA5*
**chr13**	32355212	32355212	G	C	38	*BRCA2*
**chr15**	55339305	55339305	T	-	35	*PIGB*
**chr18**	57688448	57688448	T	C	36	*ATP8B1*

Suppl. Table 3

**Chr**	**Start**	**End**	**Ref**	**Alt**	**AD**	**Gene.refGene**
**chr1**	77926770	77926770	A	G	30	*NEXN*
**chr1**	99912472	99912472	A	-	33	*AGL*
**chr1**	108807522	108807522	G	A	19	*STXBP3*
**chr2**	15478231	15478231	-	T	27	*NBAS*
**chr5**	6742542	6742542	G	A	64	*PAPD7*
**chr5**	16701358	16701358	G	A	28	*MYO10*
**chr5**	60891067	60891067	C	A	11	*ERCC8*
**chr5**	76875432	76875432	A	G	43	*S100Z*
**chr8**	100194256	100194256	G	T	31	*SPAG1*
**chr8**	142300611	142300611	C	T	42	*TSNARE1*
**chr11**	59793386	59793386	G	T	21	*STX3*
**chr13**	102872261	102872261	-	A	14	*BIVM-ERCC5;ERCC5*
**chr16**	57728490	57728490	G	A	49	*DRC7*
**chr17**	7702792	7702792	G	A	56	*WRAP53*

Suppl. Table 4

Chr	Start	End	Ref	Alt	AD	Gene.refGene
**chr1**	200409430	200409430	-	G	14	*ZNF281*
**chr2**	144429898	144429898	G	A	23	*ZEB2*
**chr3**	124329921	124329921	A	C	34	*KALRN*
**chr5**	16702573	16702574	CT	-	16	*MYO10*
**chr7**	112004099	112004099	C	T	30	*DOCK4*
**chr11**	47625713	47625713	A	G	30	*MTCH2*
**chr14**	24574332	24574332	A	T	28	*CTSG*

Suppl. Table 5

Chr	Start	End	Ref	Alt	AD	Gene.refGene
**chr1**	152761125	152761125	C	A	10	*KPRP*
**chr2**	98391949	98391949	G	A	18	*CNGA3*
**chr2**	158117928	158117928	A	-	17	*UPP2*
**chr3**	52054308	52054308	G	A	26	*DUSP7*
**chr4**	76778949	76778949	C	G	15	*SHROOM3*
**chr4**	118687939	118687939	C	T	18	*METTL14*
**chr5**	81345025	81345025	G	A	14	*ACOT12*
**chr5**	152404455	152404455	G	T	19	*NMUR2*
**chr5**	180611377	180611377	C	T	27	*FLT4*
**chr6**	99376021	99376021	A	T	24	*COQ3*
**chr7**	154991030	154991030	A	-	44	*PAXIP1*
**chr8**	100705591	100705591	A	G	16	*PABPC1*
**chr8**	134610502	134610502	C	T	15	*ZFAT*
**chr10**	97445805	97445805	C	T	41	*EXOSC1*
**chr11**	128986649	128986649	G	T	11	*ARHGAP32*
**chr12**	112876756	112876756	G	A	30	*RPH3A*
**chr14**	102012002	102012002	G	T	33	*DYNC1H1*
**chr16**	88648078	88648078	C	A	52	*CYBA*
**chr17**	73435566	73435566	C	T	56	*SDK2*
**chr17**	80099636	80099636	G	A	27	*CCDC40*
**chr22**	31712635	31712635	G	T	21	*PRR14L*

Suppl. Figure 1

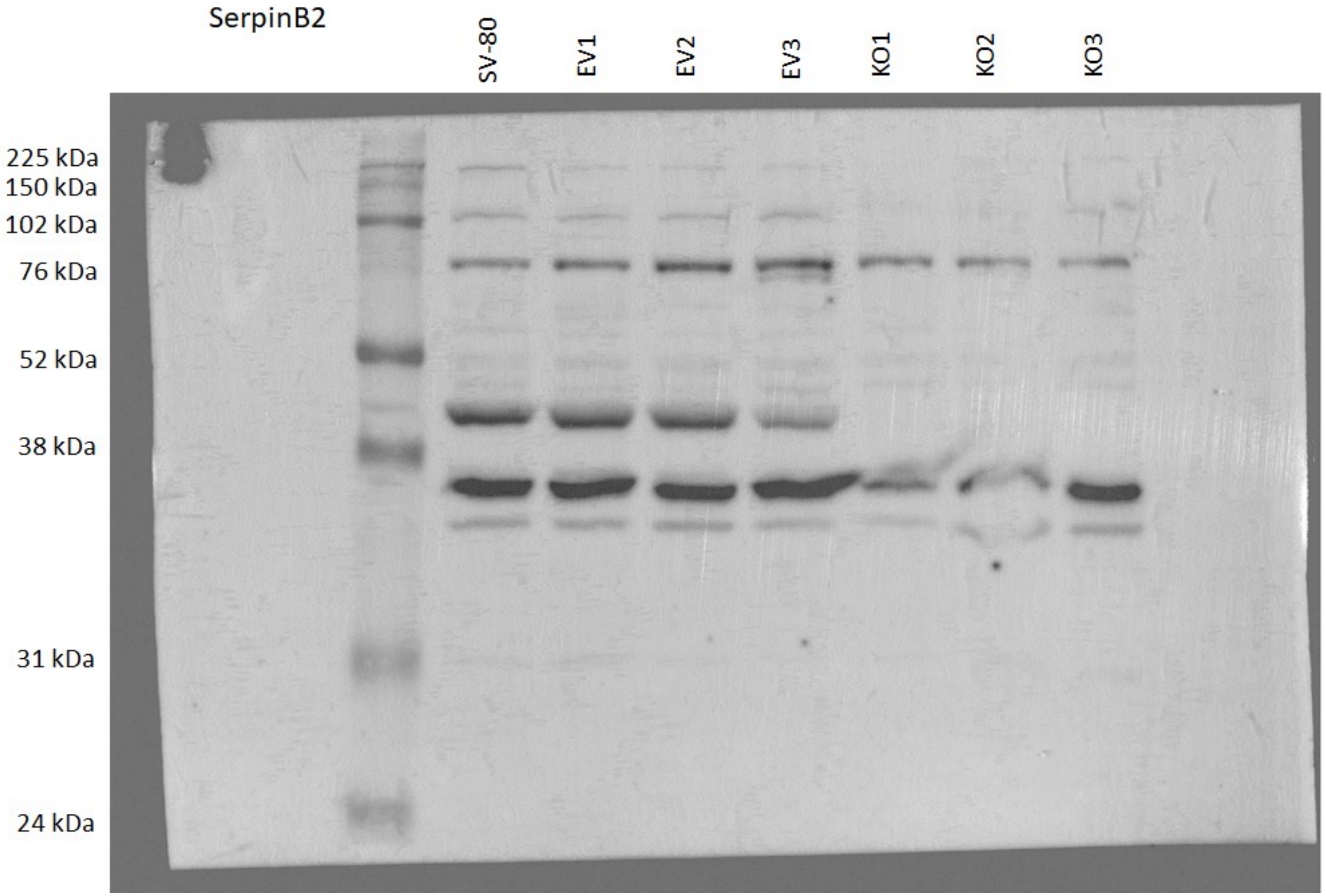

Suppl. Figure 2

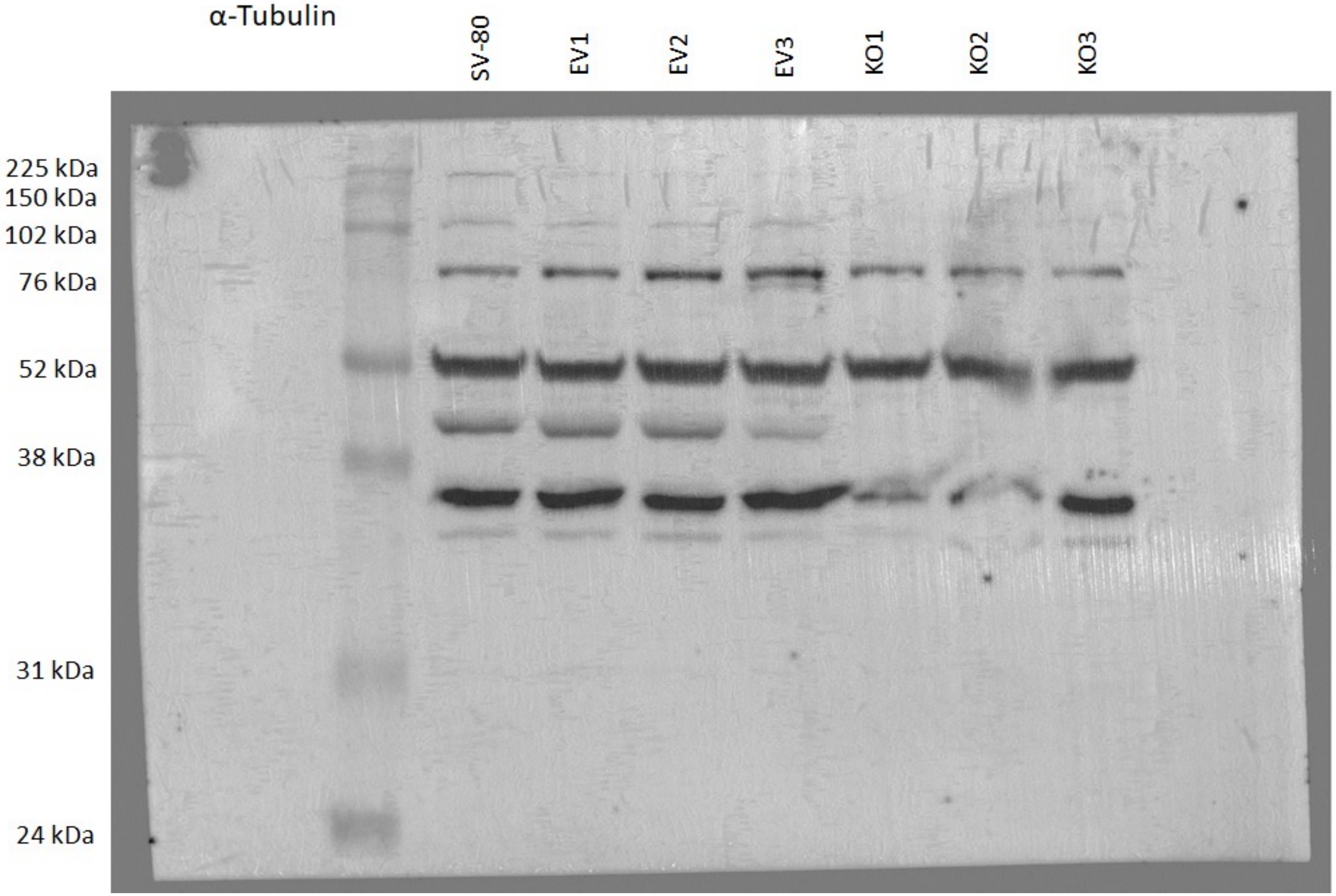

Suppl. Legends

Supl. Tab.1: List of identified genes Samples 1.3. Data of the somatic point mutations in correlation to localization, base exchange, and function. SNV: single nucleotide variant. Chr.: chromosome. Ref.: reference. Alt.: alternative. AD: coverage.

Supl. Tab.2: List of identified genes Sample 1.5. Data of the somatic point mutations in correlation to localization, base exchange, and function. SNV: single nucleotide variant. Chr.: chromosome. Ref.: reference. Alt.: alternative. AD: coverage.

Supl. Tab.3: List of identified genes Sample 1.6. Data of the somatic point mutations in correlation to localization, base exchange, and function. SNV: single nucleotide variant. Chr.: chromosome. Ref.: reference. Alt.: alternative. AD: coverage.

Supl. Tab.4: List of identified genes Sample 1.11. Data of the somatic point mutations in correlation to localization, base exchange, and function. SNV: single nucleotide variant. Chr.: chromosome. Ref.: reference. Alt.: alternative. AD: coverage.

Supl. Tab.5: List of identified genes Sample 1.13. Data of the somatic point mutations in correlation to localization, base exchange, and function. SNV: single nucleotide variant. Chr.: chromosome. Ref.: reference. Alt.: alternative. AD: coverage.

Supl. Fig.1: Western Blot with SerpinB2-AB.

Supl. Fig.2: Western Blot with alpha-Tubulin-AB.
